# Inactivation of [Fe-S] Metalloproteins Mediates Nitric Oxide-Dependent Killing of *Burkholderia mallei*

**DOI:** 10.1371/journal.pone.0001976

**Published:** 2008-04-09

**Authors:** Jessica Jones-Carson, James Laughlin, Mohammed A. Hamad, Amanda L. Stewart, Martin I. Voskuil, Andrés Vázquez-Torres

**Affiliations:** 1 Department of Microbiology, University of Colorado Health Sciences Center, Aurora, Colorado, United States of America; 2 Department of Medicine, University of Colorado Health Sciences Center, Aurora, Colorado, United States of America; Duke University Medical Center, United States of America

## Abstract

**Background:**

Much remains to be known about the mechanisms by which O_2_-dependent host defenses mediate broad antimicrobial activity.

**Methodology/Principal Findings:**

We show herein that reactive nitrogen species (RNS) generated by inducible nitric oxide (NO) synthase (iNOS) account for the anti-*Burkholderia mallei* activity of IFNγ-primed macrophages. Inducible NOS-mediated intracellular killing may represent direct bactericidal activity, because *B. mallei* showed an exquisite sensitivity to NO generated chemically. Exposure of *B. mallei* to sublethal concentrations of NO upregulated transcription of [Fe-S] cluster repair genes, while damaging the enzymatic activity of the [Fe-S] protein aconitase. To test whether [Fe-S] clusters are critical targets for RNS-dependent killing of *B. mallei*, a mutation was constructed in the NO-induced, [Fe-S] cluster repair regulator *iscR*. Not only was the *iscR* mutant hypersusceptible to iNOS-mediated killing, but its aconitase pool was readily oxidized by NO donors as compared to wild-type controls. Although killed by authentic H_2_O_2_, which also oxidizes [Fe-S] clusters, *B. mallei* appear to be resilient to NADPH oxidase-mediated cytotoxicity. The poor respiratory burst elicited by this bacterium likely explains why the NADPH oxidase is nonessential to the killing of *B. mallei* while it is still confined within phagosomes.

**Conclusions/Significance:**

Collectively, these findings have revealed a disparate role for NADPH oxidase and iNOS in the innate macrophage response against the strict aerobe *B. mallei*. To the best of our knowledge, this is the first instance in which disruption of [Fe-S] clusters is demonstrated as cause of the bactericidal activity of NO congeners.

## Introduction

The gram negative, nonmotile *B. mallei* bacillus is the causative agent of glanders, a disease that can be transmitted to humans upon cutaneous, mucosal or aerosol exposure to mucopurulent discharge from the eyes, nose and lips of infected solipeds [1]. The clinical presentation of glanders is characterized by an acute or chronic suppurative syndrome involving the upper and lower respiratory tract. If untreated, the local signs of glanders often evolve into full-blown sepsis, multiorgan system failure and shock. The poor understanding of the pathogenesis of glanders, the severity and clinical diversity of the infection and a lack of vaccines make *B. mallei* a desirable bioterrorist agent as has been demonstrated empirically in humans and animals in both World War I and II [2].

Pathogens adapted to an intracellular lifestyle avoid competition with other microbes that colonize host surfaces, while taking advantage of the nutritional resources provided by the cell. The cost of intracellular parasitism is the potential exposure to a variety of host defenses. For example, intracellular pathogens may encounter the O_2_-dependent antimicrobial mechanisms that are coupled to NADPH oxidase and iNOS enzymatic activities. The NADPH phagocyte oxidase is assembled on endocytic or plasma cell membranes, whereupon it catalyzes the NADPH-dependent univalent reduction of O_2_ to superoxide (O_2_^•−^). This radical serves as precursor to a variety of reactive oxygen species (ROS) endowed with widespread antimicrobial activity [3]. The electrogenic nature of the NADPH oxidase may also contribute to the host cell arsenal by promoting the release of cationic proteases from the proteoglycan matrix into the phagosomal lumen [4]. Professional phagocytes independently use O_2_ and NADPH in the oxidation of L-arginine with the consequent generation of L-citrulline and NO [5]. Similar to ROS, products of the reaction of NO with O_2_, O_2_^•−^ and metals are endowed with antimicrobial activity against phylogenetically diverse microorganisms [6].

Recent investigations have demonstrated that members of the genus *Burkholderia* survive within mononuclear phagocytes [7], prompting an interest in the role of ROS and RNS in defense against these bacteria [8]–[12]. The importance that the NADPH oxidase plays in resistance to some *Burkholderia* spp. is made evident by the fact that *B. cepacia* is the second most lethal infection in chronic granulomatous disease patients carrying mutations in membrane-bound or cytosolic components of the NADPH oxidase [13]. The clinical importance of NADPH oxidase in resistance to *B. cepacia* has been recreated in p47*phox*-deficient mice [14]. The NADPH oxidase has also been linked to the anti-*B. pseudomallei* arsenal of macrophages [12]. In contrast to ROS, current investigations on NO-related anti-*Burkholderia* activity have been rather controversial. Experimental animal models and macrophage cell cultures have shown that iNOS is dispensable for innate host defense against *B. cepacia* and *B. pseudomallei* [12], [14]. Moreover, others have shown that the greatest *Burkholderia* growth occurs during maximal NO synthesis [8]. Inhibition of iNOS mRNA transcription appears to underlie the lack of a role of RNS against *B. pseudomallei* [9], [10]. On the other hand, IFNγ can enhance NO-mediated intracellular killing of *B. pseudomallei* [11]. Recently, it has been reported that *B. mallei* is susceptible to NO generated by macrophages [15], although the mechanisms of antimicrobial activity remain unclear. Similarly, it remains unclear whether the NADPH oxidase plays a role in resistance to the intracellular pathogen *B. mallei*. The major goal of this study was to characterize the contribution of NADPH oxidase and iNOS hemoproteins to the macrophage antimicrobial arsenal against *B. mallei*.

## Methods

### Bacterial Strains and genetic manipulations

*B. mallei* strain ATCC 23344 was used in these studies (table 1). Bacteria grown overnight in 10 ml of Luria Bertani broth supplemented with 4% glycerol (LBG) at 37°C in a 315 RPM shaking incubator were sub-cultured into pre-warmed LBG and grown to an OD_600_ of 0.6. The *B. mallie iscR* homologue, locus BMA1709, was disrupted by inserting a 400 bp internal region of the BMA 1709 using homologous recombination. The 400 bp internal region was amplified by PCR using an *iscR* forward primer containing a BamHI restriction site (5′-tgactacggatccgcccggtgacgcttgcaggcatcag-3′) and the *iscR* reverse primer containing an SphI restriction site (5′-aagcccgtcgcatgcgcgacgggctccggcttgcgcttg-3′). After double digestion with BamHI and SphI, the PCR product was directionally cloned into the suicide vector pMO90, creating the disruption plasmid pMO126. pMO126 was introduced into *B. mallei* using triparental mating [16] in the presence of the helper *E. coli* DH5α pRK2013 strain [17]. Mating reactions were plated on LB agar plates supplemented with 30 µg/ml kanamycin to select for transconjugants, and 20 µg/ml polymyxin B to select against the helper and donor *E. coli* strains. The resulting colonies were re-streaked for isolation on LB plates supplemented with 30 µg/ml kanamycin and 20 µg/ml polymyxin B. Insertion of pMO126 into the *B. mallei* genome and the disruption of BMA 1709 was confirmed by PCR. The replicative plasmid pMO79 encoding a green fluorescence protein was conjugated into *B. mallei* strain ATCC 23344 by triparental mating as described above. The presence of pMO79 in *B. mallei* was confirmed by fluorescence microscopy.

**Table 1:** Bacterial strains and Plasmids.

Strains/Plasmids	Relevant properties	Description	Source
*B. mallei* ATCC 23344	Wild type	Isolated in 1944 from a human case of glanders: Gm^s^, Pb^r^	[50]
*B. mallei Mo126.2*	*iscR* mutant	*bma1709*::pMO126	This study
*S.* Typhimurium BC696	*fliC^−^ fljB^−^*	SL1344 Δ*fliC* Δ*fljB*	[51]
DH5α	DH5α harboring pRK2013	F^−^ ø80lacZM15 endA1 recA1 hsdR17(rK^−^ mK^+^) supE44 thi-1 ΔgyrA96 relA1Δ (lacZYA-argF)U169]	Laboratory stock
JM109	Cloning strain harboring pMO79 & pMO126	e14^−^(McrA^−^) recA1 endA1 gyrA96 thi-1 hsdR17 (rK^−^ mK^+^) supE44 relA1 Δ(lac-proAB) [F′ traD36 proAB lacI^q^ZΔM15]	Stratagene
pRK2013	Helper plasmid	RK2 derivative, Kan^r^ *mob* ^+^ *tra* ^+^ ColE1	[16]
pMO79	Mobilizable replicative plasmid	ori_pBBR1_ Kan^r^ mob^+^ *gfp rfp*	Hamad & Voskuil
pMO90	Mobilizable siucide plasmid	ColE1 Kan^r^ *mob* ^+^ *gfp xyle*	Hamad & Voskuil
pMO126	Mobilizable disruption plasmid	pMO90 carrying an internal fragment of the *B. mallei iscR* homologue	This study

Gm^s^ = gentamicin sensitive.

Pb^r^ = polymixin B resistant.

Km^r^ = kanamycin resistant.

### Macrophages

C57BL/6 and congenic *iNOS^−/−^* [18] or gp91phox^−/−^ [19] mice were bred in our animal facility according to Institutional Animal Care and Use Committee guidelines. Peritoneal macrophages were harvested from mice 4 days after intraperitoneal inoculation of 1 mg/ml sodium periodate as described [20]. The peritoneal exudate cells were resuspended in RPMI 1640 medium (Sigma-Aldrich, St. Louis, MO) supplemented with 10% heat-inactivated fetal bovine serum (BioWhittaker, Walkersville, MD), 15 mM Hepes, 2 mM L-glutamine, 1 mM sodium pyruvate (Sigma-Aldrich), and 100 U ml^−1^/100 mg ml^−1^ of penicillin/streptomycin (Cellgro) (RPMI^+^ medium). The RPMI^+^ medium used to culture the THP-1 cell line was supplemented with 50 µM 2-mercaptethanol. The peritoneal exudate cells were seeded in flat-bottom 96-well plates for macrophage killing assays at a density of 2×10^5^ cells per well. The macrophages were selected by adherence after 24 h of culture at 37°C in a 5% CO_2_ incubator. Murine J774 (clone ATCC TIB-67) and AMJ2-C11 (clone ATCC CRL-2456) and human U937 (clone ATCC CRL-1593.2) and THP1 (clone ATCC TIB-202) macrophage-like cells grown in RPMI^+^ medium supplemented as described above were used as additional sources of mononuclear phagocytes. Selected groups of macrophages were treated with 200 U/ml of IFNγ (Life Technologies, St. Paul, MN) overnight prior to *Burkholderia* infection. Just prior to infection the macrophages were washed with pre-warmed RPMI without antibiotics.

### Macrophage killing assays

The macrophage killing capacity was quantified by a gentamicin protection assay following a modified protocol described for *Salmonella* [21]. *B. mallei* were grown overnight in LBG broth and sub-cultured to OD_600_ of 0.6 as described above. The bacteria were spun down for 5 min at 1500 RPM onto the macrophages at an MOI of 200 after opsonization in 10% normal mouse serum in RPMI^+^ medium. Unless specified, extracellular bacteria were removed from the monolayers after a 2 h incubation by washing with pre-warmed RPMI^+^ medium containing 6 µg/ml of gentamicin (Sigma-Aldrich). After washing the noninternalized bacteria, the MOI was determined to be 10. *Burkholderia*-infected macrophages were lysed with 1% Triton X-100 in phosphate buffered saline (PBS) at the indicated times after challenge. The intracellular bacteria recovered at various points after infection were enumerated on LB agar plates. The % survival was calculated as (cfu t_n_/cfu t_0_)×100.

### Superoxide anion determination

O_2_^•−^ was quantified by the superoxide dismutase–inhibitable reduction of ferricytochrome c [22]. The macrophages were infected at an MOI of 40 with *Burkholderia* resuspended in phenol red–free Earle's balanced salt solution containing 60 µM ferricytochrome c. Because *B. mallei* is aflagellated, a strain of *Salmonella enterica* serovar Typhimurium deficient in *fliC fljB* was used as a positive control. After 1 h incubation in 5% CO_2_ at 37°C, the OD of the supernatants was determined spectrophotometrically at 550 nm. The concentration of O_2_^•−^ was calculated by using an ε_550_ of 2.1×10^3^ M^−1^ cm^−1^. All reagents were purchased from Sigma-Aldrich.

### Nitrite measurement

The concentration of nitrite (NO_2_^−^) produced by *Burkholderia*-infected macrophages 6 h post-challenge was estimated spectrophotometrically at 550 nm in a Biotek Synergy HT-2 reader after mixing culture supernatants with an equal volume of Griess reagent (0.5% sulfanilamide and 0.05% N-1-naphthylethylenediamide hydrochloride in 2.5% phosphoric acid). The NO_2_^−^ concentration was calculated by regression analysis using a NaNO_2_ standard curve.

### Susceptibility to RNS and ROS *in vitro*

Overnight cultures of *B. mallei* diluted 1∶100 in LBG broth were grown at 37°C with shaking to OD_600_ of 0.6. Bacteria were concentrated by centrifugation and the pellets resuspended in PBS. The bacteria were diluted 1∶100 in PBS and incubated with spermine NONOate (Cayman Chemical, Ann Arbor, MI), H_2_O_2_ (ThermoFisher Scientific, Waltham, MI), or spermine (Sigma-Aldrich, St. Louis, MO) at 37°C. At the end of the incubation, 200 units of catalase (Sigma-Aldrich, St. Louis, MO) were added to the H_2_O_2_-treated samples to consume any remaining H_2_O_2_. Serial dilutions were spotted on LBG agar plates and the number of colony forming units counted after 48 h of culture.

### Microarrays

RNA from *B. mallei* was isolated in Triazol reagent from cultures grown to an OD_600_ of 0.1 in LB medium. Selected cultures were treated with 1 mM DEANO for 30 min before the isolation of the RNA. Synthesized cDNA was labeled as previously described for *Mycobacterium tuberculosis* [23] with the following changes. An additional phenol/chloroform extraction was added to clean the RNA after isolation. The resulting RNA was further purified using a Qiagen RNeasy kit. 5 µg RNA were used for cDNA synthesis. After cDNA synthesis, the samples were further purified by phenol/chloroform extraction followed by concentration using a microcon YM-30 column (Millipore, Bedford, MA). Hybridization reactions contained 5 µl cDNA, 1 µl yeast tRNA (Sigma), and 15 µl hybridization solution (400 µl formamide, 239 µl H_2_O, 250 µl 20× SSC, 10 µl 10% SDS 10 µl). Samples were heated at 98°C for 2 min, cooled 5 min at 25°C and placed on pre-hybridized *Burkholderia* array slides (Colorado State University RM-RCE Genomics Core). Hybridization was conducted in standard hybridization chambers overnight in a 42°C water bath and washed with 1× SSC and 0.05% SDS, followed by two washes in 0.06× SSC. The slides were scanned using a Genepix Axon 4000B scanner. Initial data analysis was conducted using Genepix Pro software, followed by analysis using Microsoft Excel as described [23].

### Aconitase enzymatic assay

3 mL of stationary phase *B. mallei* grown in LBG medium were pelleted by centrifugation and resuspended in an equal volume of LB medium. Selected cultures containing 36 OD were treated with either 500 µM or 1 mM spermine NONOate for 30 min. The cultures were pelleted by centrifugation at 4°C, washed with 50 mM Tris-HCl buffer, pH 7.6 and the cytoplasmic proteins extracted in 400 µL B-PER (Pierce, Rockford IL) after vortexing for 30 sec. The samples were diluted 1∶2 in 50 mM Tris-HCL buffer, pH 7.6. After removing the insoluble fraction by centrifugation, the supernatants were filter-sterilized. Aconitase activity in the soluble fractions was estimated spectrophotometrically at 240 nm by following the formation of cis-aconitate in 50 mM Tris-HCl buffer pH 7.4 containing 30 mM isocitrate. Aconitase activity is expressed as ΔOD_240_/min/µg protein.

### Electron microscopy

The phagocytes were plated as described above for killing assays at a density of 4×10^5^ per chamber of a 8-well Permanox Labtek chamber slide system (Nalgene Nunc International, Rochester, NY). The macrophages were challenged with *B. mallei* at an MOI of 10 as described above. Between 2 and 3.5 h after infection, the cells were fixed in 2.5% glutaraldehyde in phosphate buffer, pH 7.4. The specimens were postfixed in 1% osmium tetraoxide, treated with uranyl acetate, dehydrated in ascending ethanol series, and infiltrated with Embed 812. Ultrathin sections were examined in a FEI Technai 62 electron microscope operated at 80 kV.

### Fluorescence microscopy

Macrophages plated on 13 mm glass coverslips in a 24-well plate at a density of 2.5×10^5^ cells/well were infected as described above with GFP-expressing *B. mallei*. The specimens were washed in PBS 3 h after infection and fixed overnight at 4°C in 2% paraformaldehyde (Electron Microscopy Sciences, Hatfield, PA) diluted in PBS. After fixation, the coverslips were washed in PBS and the cells were permeablized for 5 min with 0.1% Triton X-100 in PBS. After washing, F-actin was visualized by labeling the infected macrophages with Alexa Fluor 568 phalloidin (Invitrogen). The coverslips were washed with PBS and mounted onto glass slides prepped with Vectashield mounting medium containing DAPI nucleic acid stain (Vectashield Laboratory, Burlingame, CA). The specimens were examined with a Zeiss Axiophot fluorescence microscope equipped with a charge-coupled-device camera controlled by the SlideBook deconvolution image-processing software (Intelligent Imaging Innovations, Denver, CO).

### Statistical analysis

Data are presented as mean ± SEM or in box-and-whiskers plots as median, intraquartile and total ranges. A two-tailed, Student's *t*-test was used for statistical analysis and the data considered statistically significant when *p*<0.05.

## Results

### Survival of *B. mallei* in human and murine cell lines

**Figure 1:**
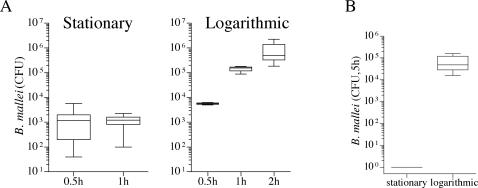
Antimicrobial activity of murine and human macrophage-like cell lines against *B. mallei*. Intracellular survival of *B. mallei* was studied over a 20 h period in murine (i.e., J774 and AMJ) and human (i.e., THP1 and U937) macrophage-like cell lines. Data from 8–14 independent observations gathered in 3 separate experiments are represented in box-and-whiskers plots as median, intraquartile and total ranges.

The number of log phase *B. mallei* internalized by J774 cells increased as a function of time, reaching approximately 10^6^ CFU/10^5^ macrophages 2 h after infection. Because of this delay phagocytosis, all subsequent *Burkholderia* infections, unless indicated, were carried out for 2 h before gentamicin was added to the medium. Mice offer a wide variety of genetic reagents that facilitate investigations of O_2_-dependent host defenses in host–pathogen interactions. Therefore, we deemed it important to compare the intracellular growth of *B. mallei* in a number of murine (J774 and alveolar AMJ-C11 macrophages) and human (i.e., THP1 and U937) cell lines (fig. 1). Remarkably, all murine and human macrophage-like cell lines tested killed *B. mallei* with very similar kinetics. These findings indicate that murine macrophages are a relevant model in which to study *B. mallei*-phagocyte interactions.

### IFNγ enhances killing of *B. mallei* by macrophages

**Figure 2:**
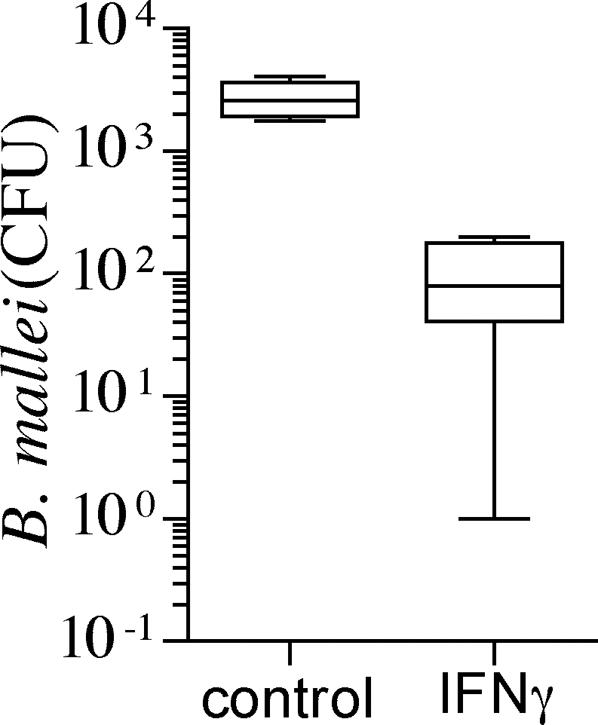
Anti-*Burkholderia* activity of IFNγ-primed macrophages. The intracellular survival of *B. mallei* was assessed in control and IFNγ-primed J774 cells. Selected groups of macrophages were treated with 200 U/ml IFNγ 16 h before infection. Data from 6 independent observations gathered on 2 separate experiments are represented in box-and-whiskers plots as median, intraquartile and total ranges. *P<0.01 compared to unstimulated control macrophages.

It has been shown that IFNγ enhances the antimicrobial activity of macrophages against the opportunistic pathogen *B. pseudomallei*. However, to our knowledge, no data are available on the role that IFNγ plays in intracellular resistance to *B. mallei*. Because IFNγ has recently been shown to be critical for resistance of mice to *B. mallei* [24], we tested the effect of IFNγ treatment on the anti-*Burkholderia* activity of J774 cells (fig. 2). IFNγ-primed macrophages were significantly (p<0.01) more efficient at killing *B. mallei* than unstimulated controls. The number of *B. mallei* recovered from IFNγ-treated macrophages was at least 10-fold lower than controls (fig. 2). In some cases, IFNγ-primed J774 cells completely eliminated the bacteria from the cell cultures. These data demonstrate that IFNγ enhances the innate resistance of murine macrophages to *B. mallei*.

### Reactive oxygen and nitrogen species differentially contribute to the anti-*B. mallei* activity of IFNγ-primed macrophages

**Figure 3:**
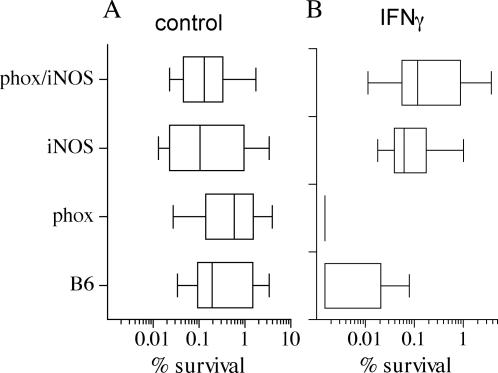
Contribution of reactive oxygen and nitrogen species to the anti-*Burkholderia* activity of macrophages. The contribution of ROS and RNS to host defense against *B. mallei* was assessed by comparing the antimicrobial activity of primary macrophages isolated from immunocompentent C57BL/6 mice (control) or congenic phox^−/−^, iNOS^−/−^ or phox^−/−^iNOS^−/−^-immunodeficient mice. Selected groups of macrophages were treated with 200 U/ml IFNγ 16 h before infection. The number of *B. mallei* recovered from the macrophages was estimated 5 h after the infected macrophages were treated with gentamicin. Data from 16 independent observations gathered on 3 separate experiments are represented in box-and-whiskers plots as median, intraquartile and total ranges. *P<0.05 compared to unstimulated control macrophages. **P<0.05 compared to IFNγ-primed macrophages from C57BL/6 mice.

ROS and RNS are key components of the antimicrobial arsenal of the innate and the activated response of macrophages against a variety of intracellular pathogens [25]. To test the importance of ROS and RNS in the killing of *B. mallei*, macrophages were obtained from C57BL/6 mice and their congenic gp91*phox* ^−/−^, iNOS^−/−^ and gp91*phox* ^−/−^/iNOS^−/−^ immunodeficient strains. Unstimulated macrophages isolated from C57BL/6 mice killed over 99% of *B. mallei* within 5 h of infection (fig. 3A). Strikingly, singly or doubly immunodeficient macrophages lacking gp91*phox* and/or iNOS hemoproteins exhibited similar bactericidal activity as that of unstimulated wild-type controls. These findings suggest that neither ROS nor RNS appear to play a significant role in the early killing of *B. mallei* by unstimulated macrophages. Similar to the improved killing seen in the J774 cell line, IFNγ increased (p<0.001) the anti-*B. mallei* activity of primary macrophages isolated from C57BL/6 mice. It should be noted that some of the IFNγ-primed macrophages completely eliminated *B. mallei* from the cultures, a phenomenon that was seen in all of the IFNγ-primed, gp91*phox*-deficient macrophages (fig. 3B). These findings suggest that ROS are dispensable for the anti-*B. mallei* activity of IFNγ-primed macrophages. Conversely, RNS contributed to the anti-*B. mallei* arsenal of IFNγ-activated macrophages (fig. 3B). In fact, this intracellular pathogen was recovered in significantly higher numbers from both IFNγ-primed, iNOS^−/−^ and iNOS^−/−^/gp91*phox* ^−/−^ macrophages than from wild-type or gp91*phox* ^−/−^ controls. The killing activity of IFNγ-treated, iNOS- or iNOS/gp91*phox*- deficient macrophages was similar to that of unstimulated wild-type controls. Collectively, these data indicate that RNS but not ROS mediate the anti-*Burkholderia* activity of IFNγ-primed macrophages, while neither RNS nor ROS appear to add to the early innate host defenses of macrophages against *B. mallei*.

### *B. mallei* is susceptible to RNS

**Figure 4:**
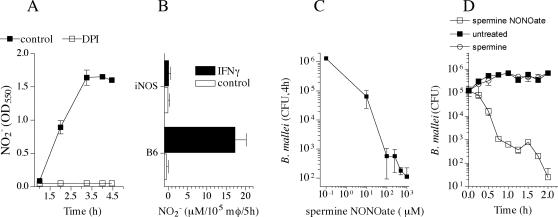
Antimicrobial activity of reactive nitrogen species generated by *Burkholderia*-infected macrophages. The ability of *B. mallei* to generate NO_2_^−^ in RPMI^+^ medium is shown in A. Selected samples were treated with 10 µM of the flavin inhibitor diphenyleneiodonium (DPI). The NO_2_^−^-producing capacity of macrophages responding to *B. mallei* was assessed in low NO_3_^−^ DMEM medium (B). The survival of *B. mallei* to increasing concentrations of the NO donor spermine NONOate is shown in C. The effects of 100 µM spermine NONOate on the viability of *B. mallei* was studied overtime (D). Untreated or spermine-treated *B. mallei* were used as controls. Data in panel A are represented in box-and-whiskers plots as median, intraquartile and total ranges. The rest of the data represent the mean ± sem.

The poor anti-*Burkholderia* activity of IFNγ-primed macrophages lacking iNOS suggest that RNS generated by activated phagocytes exert early antimicrobial activity against *B. mallei*. Consequently, the ability of macrophages to produce NO in response to *B. mallei* infection was tested by measuring the accumulation of NO_2_^−^. Supernatants of *B. mallei*-infected J774 cells contained about 200 µM NO_2_^−^ after 5 h of infection (fig. 4A). The concentration of NO_2_^−^ seen in *B. mallei*-infected cells closely paralleled the NO_3_^−^ levels contained in RPMI medium, raising the possibility that a bacterial nitrate reductase activity is responsible for the observed NO_2_^−^ burst. This idea was substantiated by the fact that the putative *B. mallei* nitrate reductase activity could be inhibited by the flavoprotein inhibitor diphenyleneiodonium (fig. 4A). To determine whether host cell iNOS activity can be stimulated upon *B. mallei* challenge, the capacity of macrophages to generate NO_2_^−^ was therefore estimated in NO_3_^−^-free medium. As shown in figure 4B, IFNγ-primed macrophages, but not unstimulated controls, generated copious amounts of NO congeners in response to *B. mallei*. Accumulation of NO_2_^−^ in the supernatants reflects a functional iNOS enzymatic complex, since the amount of NO_2_^−^ generated by iNOS-deficient macrophages was negligible. Figure 4C shows that *B. mallei* are extraordinarily susceptible to RNS. Spermine NONOate reduced the viability of *B. mallei* in a dose and time dependent manner (fig. 4C & D). The viability of *B. mallei* was significantly reduced after a 4 h exposure to as low as 10 µM of the NO donor spermine NONOate (fig. 4C). A time course indicated that the killing of *B. mallei* by 100 µM spermine NONOate was already evident after 30 min and continued unabated for at least 2 h (fig. 4D). The anti-*B. mallei* effects associated with spermine NONOate are specific to RNS generated by this NO donor, since the viability of *B. mallei* was similarly unaffected upon culture in PBS or exposure to the spermine base control.

### NO enhances transcription of genes associated with [Fe-S] cluster assembly

**Figure 5:**
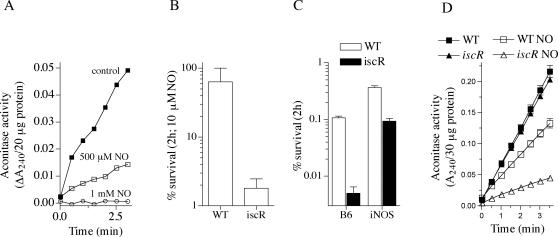
*iscR*-deficient *B. mallei* is hypersusceptible to NO-mediated cytotoxicity. The aconitase activity of *B. mallei* exposed to spermine NONOate (NO) for 30 min can be seen in panel A. Panel B shows the cytotoxicity of 10 µM spermine NONOate against wild-type (WT) or *iscR*-deficient *B. mallei*. The intracellular survival of WT and isogenic *iscR*-deficient *B. mallei* was recorded in IFNγ-treated macrophages isolated from wild-type C57BL/6 mice (B6) or iNOS-deficient congenic controls (C). The % survival after 2 h of infection was estimated according to the number of bacteria originally added to the macrophages. The aconitase activity present in overnight cultures of wild-type or *iscR*-deficient *B. mallei* is shown in D. Selected groups of bacteria were treated with 500 µM spermine NONOate for 30 min before the aconitase activity present in cytoplasmic extracts was recorded. Panels A & D represent data of two independent experiments. Data in panel B & C are the mean ± sem from 4–8 independent observations.

To gain insight into the targets of RNS antimicrobial activity, global transcriptional profiles were compared between *B. mallei* cultures untreated or treated with sublethal NO concentrations. Microarray expression analyses showed the transcriptional up-regulation of gene products involved in iron acquisition and [Fe-S] cluster assembly and repair (table 2). This pattern of transcription suggests that [Fe-S] clusters of dehydratases are critical *B. mallei* targets of RNS-mediated cytotoxicity. Accordingly, the enzymatic activity of the [Fe-S] cluster protein aconitase was markedly reduced in *B. mallei* cultures treated with spermine NONOate. For instance, about 100 pmoles of NO per 10^6^ bacteria completely inactivated the activity of aconitase (fig. 5A). To test more directly whether [Fe-S] clusters are a key target of the anti-*B. mallei* activity of RNS, an insertion mutant was constructed in the *iscR* transcriptional factor that regulates [Fe-S] cluster assembly. The lack of *iscR* increased the susceptibility of *B. mallei* to RNS generated by the NO donor spermine NONOate (fig. 5B). We next tested whether the *iscR* mutant is susceptible to NO produced by activated macrophages. Because wild-type *B. mallei* is already highly susceptible to the iNOS-mediated antibacterial activity of IFNγ-primed macrophages after 5 h of infection (fig. 3B), the survival of wild-type and *iscR*-deficient *B. mallei* was compared after 2 h of challenge. As anticipated (fig. 5B), at this early time the *iscR* mutant was more susceptible than wild-type controls to the antimicrobial activity of IFNγ-primed macrophages (fig. 5C). The difference in intracellular survival of wild-type and *iscR*-deficient bacteria was not as great in IFNγ-primed macrophages lacking iNOS compared to those containing iNOS (fig. 5C). These data suggest that *iscR* mutant bacteria are hypersusceptible to the RNS generated by the host cells. The aconitase activity of the *iscR* mutant grown overnight in LBG medium was similar to that of wild-type controls (fig. 5D). However, the enzymatic activity of aconitase was more vulnerable to RNS-mediated inactivation in the absence of a functional *iscR*. Together, these findings are consistent with the notion that [Fe-S] clusters are key targets of the RNS-dependent bactericidal activity against *B. mallei*.

**Table 2:** Iron-sulfur cluster assembly genes induced by nitric oxide.

Gene No.	Gene	Ratio	St.Dev.	Protein Function	Pathway
BMA2033		2.7	1.4	iron compound ABC transporter	iron assimilation
BMAA0021		4.6	1.3	siderophore-interacting protein	iron assimilation
BMAA0883		4.5	2.7	iron permease, FTR1 family	iron assimilation
BMAA1800		14.1	3.0	putative bacterioferritin-associated ferredoxin	iron assimilation
BMA0666	*cysD-1*	3.5	0.7	sulfate adenylyltransferase, subunit 2	sulfur assimilation
BMA0667	*cysN*	4.3	0.5	sulfate adenylyltransferase, subunit 1	sulfur assimilation
BMA2729		2.2	0.4	sulfur carrier protein	sulfur assimilation
BMA0610	*sufS*	3.8	1.7	cysteine desulfurase	[Fe-S] assembly
BMA1706		3.3	0.1	[Fe-S] assembly accessory protein	[Fe-S] assembly
BMA1707	*iscU*	2.3	0.7	[Fe-S] assembly scaffold	[Fe-S] assembly
BMA1708	*iscS-1*	3.9	0.5	cysteine desulfurase	[Fe-S] assembly
BMA1709	*iscR*	6.9	2.9	[Fe-S] assembly transcription factor	[Fe-S] assembly
BMAA1430		2.0	0.5	[Fe-S] binding protein	[Fe-S] assembly

### *B. mallei* is resistant to the oxidative burst of macrophages despite its marked susceptibility to ROS

**Figure 6:**
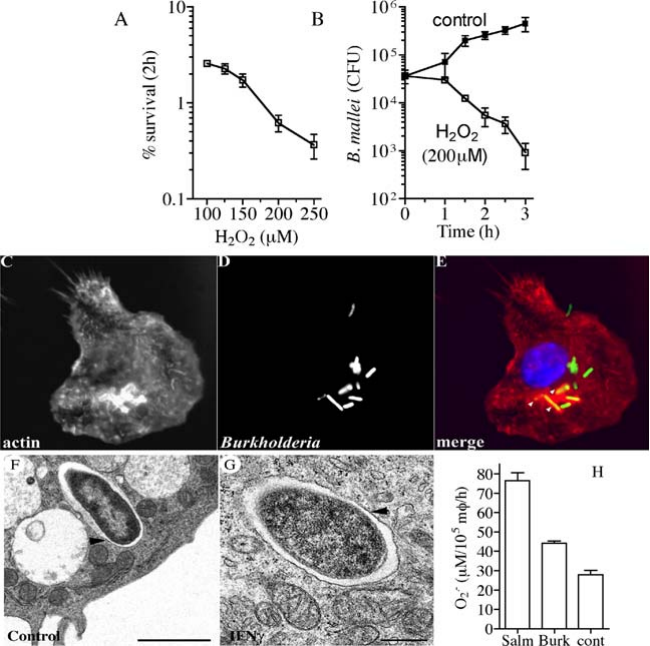
Susceptibility of *Burkholderia* to ROS generated by macrophages. The susceptibility of *B. mallei* to increasing concentrations of H_2_O_2_ is shown in A. Kinetic analysis of the viability of *B. mallei* in response to 200 µM H_2_O_2_ is shown in B. The cellular localization of phalloidin-labeled actin filaments (C) and GFP-expressing *B. mallei* (D) was examined by fluorescence microscopy. The host cell nucleus labeled with DAPI (blue), *B. mallei* (green) and actin (red) are visualized in the merge panel (E). The micrograph is representative of a total of 285 bacteria examined from 33 cells collected in 2 independent experiments. The location of *B. mallei* in the cytoplasm of control (F) and IFNγ-primed (G) macrophages from C57BL/6 mice was visualized by transmission electron microscopy 3.5 h after infection. Bars in F and G denote 2 and 0.5 µm, respectively. The micrographs are representative of 97 *Burkholderia*-containing phagosomes from 5 independent experiments. Production of superoxide by *B. mallei*-infected macrophages isolated from C57BL/6 mice was assessed spectrophotometrically as the superoxide dismutase-inhibitable reduction of cytochrome c (H). Data in panels A, B and G represent the mean ± sem from 6 independent observations.

We investigated the possible mechanism for the apparent dispensability of the NADPH oxidase in the intracellular killing of *B. mallei*. *B. mallei* was found to be susceptible to authentic H_2_O_2_ (fig. 6A). As little as 100 µM H_2_O_2_ killed over 90% of *B. mallei* after 2 h. A kinetic study revealed that the viability of *B. mallei* was markedly reduced 90 min upon exposure to 200 µM H_2_O_2_, declining further thereafter (fig. 6B). These findings demonstrate that *B. mallei* are extraordinarily susceptible to ROS-mediated killing. The ability of *Burkholderia* to escape into the cytosol [26], [27] could rationalize the apparent dispensability of NADPH oxidase in the anti-*B. mallei* arsenal of macrophages. To test the intracellular localization of *B. mallei*, the ability of the bacterium to form actin-tails was evaluated by fluorescence microscopy. As shown in figure 6C–E, *B. mallei* do not appear to polymerize actin tails at the early times examined here, although polymerization of actin was evident in about 10% of the *B. mallei*-containing phagosomes (figure 6E). Electron microscopy studies revealed that the totality of *B. mallei* were found within phagosomes in both unstimulated and IFNγ-primed macrophages (fig. 6F and G). Collectively, the fluorescence and electron microscopy studies indicate that *B. mallei* remain within the confines of phagosomal membranes during the early stages of infection studied herein, irrespective of whether the phagocytes are stimulated with IFNγ (fig. 6F & G). We tested whether the seeming dispensability of the NADPH oxidase in the anti-*B. mallei* activity of macrophages was due to a poor respiratory burst. Estimates of O_2_^•−^ production by the superoxide dismutase-inhibitable reduction of cytochrome c revealed that *B. mallei* was a poor stimulant of the respiratory burst as compared to an aflagellated *fliC fljB Salmonella* control (fig. 6H).

## Discussion

O_2_-dependent host defenses are the best-characterized components of the antimicrobial arsenal of professional phagocytes. O_2_ is used by NADPH oxidase and iNOS hemoproteins for the generation of O_2_^•−^ and NO, which are the precursors of a plethora of ROS and RNS. Pathogenic microorganisms show a spectrum of susceptibilities to the antimicrobial actions of RNS. For example, RNS are essential for resistance to *Mycobacterium tuberculosis* and *Leishmania major* [12], [28], but appear to be dispensable in host defense against *Legionella pneumophila* and *Pseudomonas aeruginosa* [25]. Similar to *Mycobacterium* and *Leishmania*, our investigations have shown that the intracellular pathogen *B. mallei* is highly susceptible to RNS-mediated host defenses. We find that the inactivation of [Fe-S] clusters is a major component of the anti-*B. mallei* activity of RNS.

Poor killing of *B. mallei* by IFNγ-treated macrophages lacking iNOS, irrespective of the NADPH oxidase status of the cell, suggests that the RNS-mediated killing of *B. mallei* does not depend on host-derived ONOO^−^ synthesis. NO itself, nitrogen oxides derived from the autooxidation of NO in the presence of O_2_, or ONOO^−^ generated in the bacterial cytoplasm from the reaction of NO with endogenous O_2_^•−^ represent a few of the RNS that may be responsible for the iNOS-dependent killing of *B. mallei*. *B. mallei* are extraordinarily susceptible to RNS as shown by the profound killing seen after treatment with 10 µM spermine NONOate. NO donors also exert antimicrobial activity against phylogenetically diverse microbes such as *Candida albicans*, *Salmonella enterica* and *Escherichia coli* [29]–[31]. However, NO donors, even when used at high micromolar or low millimolar concentrations, are cytostatic for these microbial pathogens. Therefore, our studies identify *B. mallei* as one of the few pathogens for which host-derived RNS are microbicidal.

Aconitase is inactivated upon incubation of *B. mallei* with NO congeners, possibly reflecting the RNS-dependent oxidation of the solvent exposed Fe_α_ in the [Fe-S] cluster [32], [33]. It is therefore not surprising that genes encoding for iron-sulfur cluster assembly are highly transcribed in NO-treated *B. mallei*. RNS-mediated oxidation of [Fe-S] clusters of dehydratases [33]–[36] has for a long time been seen as a concomitant event of cytotoxic, NO-producing macrophages [32]. However, a causative relationship between the oxidation of [Fe-S] clusters and killing by RNS has not been previously demonstrated. The rapid loss in viability and aconitase activity of an *iscR* mutant exposed to sublethal NO concentrations demonstrate a direct relationship between [Fe-S] cluster damage and the bactericidal activity of RNS against *B. mallei*. Oxidation of [Fe-S] prosthetic groups of dehydratases critical for intermediate metabolism may explain the bactericidal activity of RNS against the obligate aerobe *B. mallei*. According to our model, the overt resistance of facultative anaerobes to NO reflects the ability of these microbes to generate energy by switching from oxidative phosphorylation heavily dependent on RNS-modifiable dehydratases to fermentative pathways resilient to nitrosative stress. Future experiments will be needed to test this hypothesis.

Our data indicate that the NADPH oxidase is dispensable for intracellular killing of *B. mallei*. These findings might seem striking since *B. mallei* are readily killed upon exposure to H_2_O_2_. Microbes have devised an assortment of strategies to avoid NADPH oxidase-dependent cytotoxicity. For example, detoxification of oxyradicals by superoxide dismutases, catalases and hydroperoxydases or scavengers such as low molecular weight thiols are efficient ways to diminish oxidative stress [6]. The antimicrobial activity of the NADPH oxidase is best manifested against intracellular pathogens confined within endocytic membranes [37], [38]. At the early times examined here, *B. mallei* do not escape into the cytosol as has been shown for various *Burkholderia* spp. at later time points after infection [27], [39]. The poor respiratory burst elicited by *B. mallei* represents a mechanism for minimizing exposure to the NADPH oxidase during the time that this intracellular pathogen remains in the phagosome. Analogous to other microorganisms [40], the putative polysaccharide extracellular capsule [41] may help *B. mallei* avoid signaling cascades that stimulate a productive respiratory burst. The resistance of *B. mallei* to the NADPH oxidase-mediated antimicrobial activity of macrophages does not, however, eliminate the possibility that this hemoprotein may play a role in the purulent phase of the host response as has been shown for *B. cepacia* and *B. pseudomallei* [12], [14].

O_2_-independent mechanisms are a significant component of the innate killing of *B. mallei* as indicated by the fact that macrophages from doubly immunodeficient mice lacking both NADPH oxidase and iNOS hemoproteins efficiently kill this bacterium. Innate activation of the autophagic pathway as described for the respiratory pathogen *Legionella pneumophila* [42]–[44] is one possible mechanism for the O_2_-independent killing of *B. mallei* seen in macrophages lacking both gp91*phox* and iNOS. Recent studies have shown that small GTPases induced in response to IFNγ stimulate killing of *M. tuberculosis* by promoting autophagosome formation [45], [46]. Most of the enhanced antimicrobial activity stimulated by IFNγ against *M. tuberculosis* and *Salmonella enterica* is nonetheless associated with the production of RNS [21]. In contrast to these two intracellular pathogens, the enhanced killing of *B. mallei* by IFNγ-primed macrophages is exclusively dependent on the expression of functional iNOS. This role for RNS in the bactericidal activity of IFNγ-primed macrophages against *B. mallei* contrasts with the apparent dispensability of iNOS to the early innate host defenses of unstimulated macrophages. The short time course of the infection studied herein likely prevented innate production of nitrogen oxides in *B. mallei*-infected macrophages. Given the extraordinary susceptibility of *B. mallei* to RNS (studies herein) and the stimulation of TLR4 signaling by LPS purified from *B. mallei* [47], it is not surprising that NO produced at later times of the innate response contributes to the killing of this intracellular pathogen [15].

In summary, our studies indicate that despite its susceptibility to ROS and RNS, *B. mallei* are selectively killed by RNS generated by IFNγ-activated macrophages. These investigations place *B. mallei* among the limited number of microorganisms for which NO congeners are microbicidal. Our biochemical and genetic studies indicate that [Fe-S] clusters are critical targets of RNS-mediated killing of *B. mallei*. The extraordinary hypersusceptibility of *B. mallei* to RNS raises the intriguing possibility that NO donors could be explored as antibiotics to treat glanders, as shown in the experimental treatment of cutaneous mycosis and leishmaniasis [48], [49]. However, analogous to herpes simplex virus and toxoplasma (reviewed in 49), the possibility exists that despite exerting potent antimicrobial activity, NO could still exacerbate pathology. Future experiments will be needed to determine the value of NO donors in the treatment of glanders.
